# Infraorbital Swelling Induced by an Inadvertent Local Anesthesia Technique in a Six-Year-Old Female Child: A Clinical Case Report

**DOI:** 10.7759/cureus.85748

**Published:** 2025-06-11

**Authors:** Amilia Elizabeth, Rathina Venkateswaran, Parisa N Baghkomeh, Navami Gopan, Sreni K S

**Affiliations:** 1 Department of Pedodontics and Preventive Dentistry, Meenakshi Ammal Dental College and Hospital, Meenakshi Academy of Higher Education and Research, Chennai, IND

**Keywords:** dental care, extraction, local anesthesia, needlestick injury, pediatric dentistry

## Abstract

Local anesthesia (LA) plays a crucial role in managing pain during dental procedures, but its administration must be handled with care, particularly in children, where complications are not uncommon. This report presents a case involving a six-year-old girl who had a broken upper right first molar due to decay for the past three months. An extraction was planned under LA. Following the infiltration technique of LA administration using 2% lignocaine solution with 1:80,000 adrenaline, swelling appeared in the right infraorbital region almost immediately. The swelling resulted from inadvertent deposition of the anesthetic in the infraorbital area due to excessive needle penetration, which occurred as the child became uncooperative during the injection. The patient was prescribed analgesic eye drops and a topical ophthalmic ointment by the ophthalmologist, and the symptoms started subsiding within four hours. This report also explores prevention and management strategies for such complications in pediatric dental care.

## Introduction

Local anesthesia (LA) eliminates or drastically reduces nociceptive impulses, temporarily reducing the pain sensations during dental procedures [1]. LA is defined as a loss of sensation in a circumscribed area of the body caused by a depression of excitation of nerve endings or inhibition of the conduction process in the peripheral nerves [2]. Intraoral LA injections are relatively safe procedures that commonly involve drug administration near a terminal nerve branch or a nerve trunk [3]. Patient evaluation, tissue preparation, and administration techniques may reduce the local and systemic complications associated with LA [4]. Younger children are at a greater risk of experiencing adverse drug events. It is essential for the pediatric dentist to consider the appropriate dosage of LA based on the child's body weight. This helps mitigate the risk of toxicity and ensures that the anesthesia duration is optimal. Proper needle usage and injection techniques are crucial for providing safe and comfortable dental care to young patients.

Knowledge of the anatomy of the head and neck allows for precise depth of penetration and deposition of the anesthetic solution and helps minimize complications. Adverse events such as swelling or hematoma may occur following the administration of LAs due to traumatic injection techniques, allergies to the anesthetic constituents, a needle inadvertently puncturing a blood vessel, or the administration of an irritating solution [5].

In dental LA, eye signs occasionally occur as a rare complication, affecting approximately one in 1,000 cases, and are often unreported [6]. This unique report emphasizes an ocular complication following dental anesthesia in a six-year-old girl, underscoring the importance of vigilant and systematic patient management.

## Case presentation

The mother of a six-year-old girl presented to the Department of Pediatric and Preventive Dentistry with the chief complaint of a broken, sharp, and decayed upper right first molar, present for the past three months. The patient reported no history of pain or swelling. According to her mother, there was no relevant medical history. Additionally, the mother mentioned that the child had undergone restoration and pulpectomy under LA on her lower right molars two days prior.

**Figure 1:**
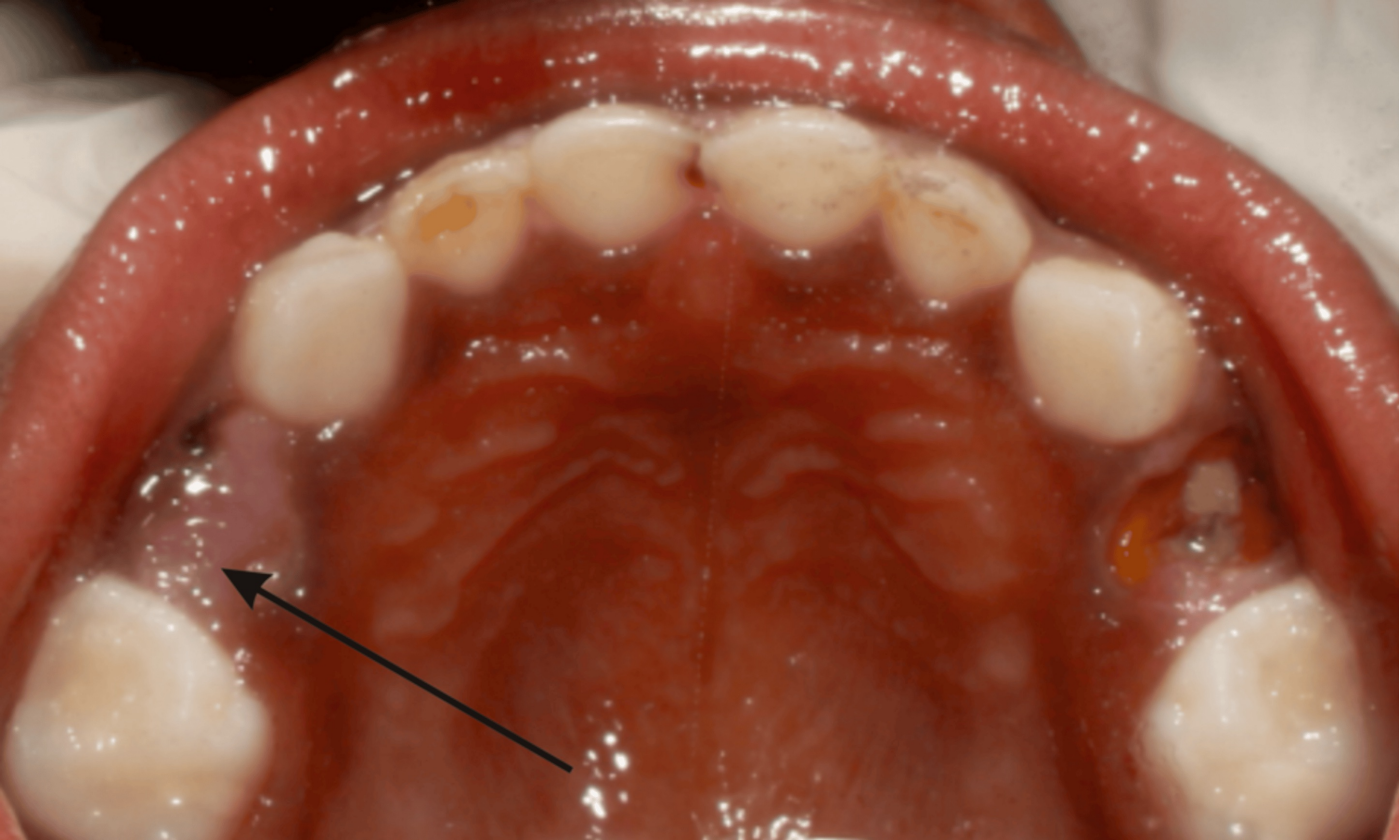
Preoperative photograph of the maxilla, root stump-54 The arrow mark shows the retained roots of 54, which is indicated for extraction under local anesthesia

**Figure 2:**
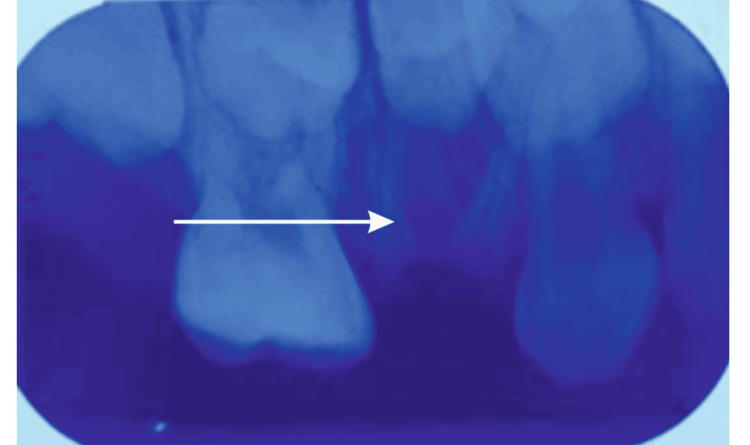
Intraoral periapical (IOPA) radiograph of 54 The IOPA of 54 reveals a radiopaque root-like structure suggestive of mesial, distal, and palatal root stumps of 54

The general examination revealed no abnormal findings, and the extraoral assessment showed no facial asymmetry. Intraoral examination revealed the presence of retained roots of tooth 54, resulting from untreated dental caries (Figure 1). This clinical finding was confirmed by a radiographic investigation using an intraoral periapical (IOPA) radiograph of 54 (Figure 2). In the radiograph, gross destruction of the crown structure was evident along with mesial, distal, and palatal radicular radiopacities suggestive of retained roots in the 54 region. The IOPA radiograph also revealed an underlying developing tooth bud of 14. The surrounding bone in relation to 54 appeared to be normal. From the investigations, a final diagnosis of retained roots of 54 due to untreated dental caries was made.

The treatment plan involved extracting the retained roots of 54, followed by the placement of a space maintainer. Informed consent was obtained from the patient’s mother, and the extraction was scheduled under LA.

The injection site was cleaned with sterile gauze, and a topical anesthetic gel (LOX 2% lignocaine hydrochloride) was applied to the buccal vestibular region to prepare for the extraction of the retained roots of 54. After one minute, 0.8 mL of LA solution (LIGNOX^®^ 2%, Indoco Remedies Limited, Mumbai) as buccal infiltration was administered.

The child's initial behavior, as evaluated using Wright’s Behavior Assessment, was classified as Rating No. 3: positive (+)-tense-cooperative. Initially, the child accepted the procedure and followed the dentist’s instructions, although with noticeable hesitation and caution [7]. LA was administered following the Tell-Show-Do behavior management technique. However, during the needle prick and LA deposition, the child became hysterical and uncooperative, causing the operator to lose control of the needle. A 24-gauge, 1-inch (0.55 × 25 mm) needle was used for the procedure. Due to the child’s sudden movement, the needle deviated from its intended path and advanced toward the infraorbital region, where approximately 1 mL of LA solution was inadvertently deposited.

**Figure 3:**
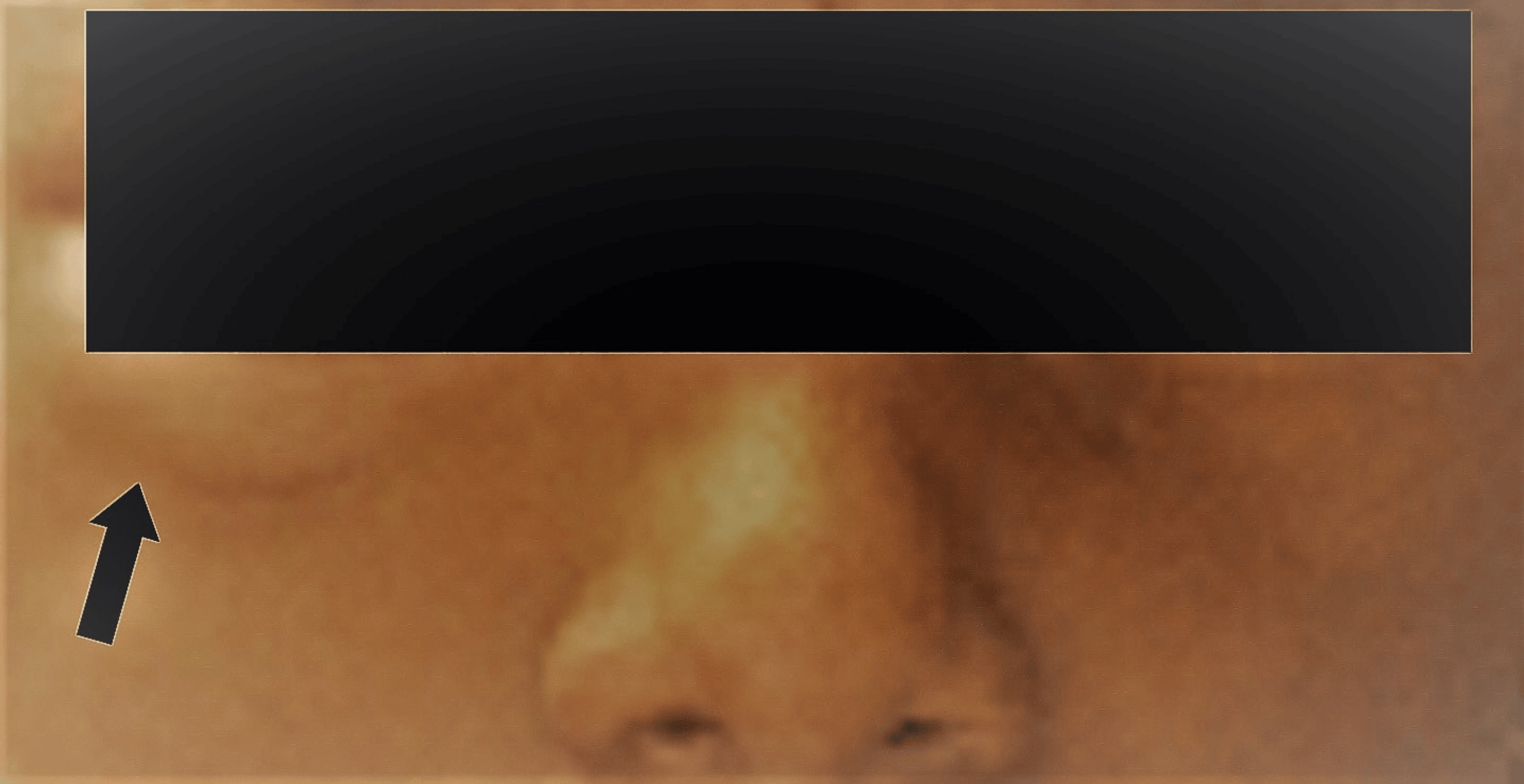
Extraoral photograph with infraorbital swelling immediately after LA deposition The arrow mark indicates the area with mild infraorbital swelling immediately after LA deposition LA: local anesthesia

A swelling immediately appeared in the right infraorbital region (Figure 3). Upon inspection, a single, diffuse, semilunar-shaped swelling measuring approximately 1.5 × 0.5 cm was observed. It was confined to the area between the right lower eyelid and the infraorbital ridge in a superoinferior direction and extended mediolaterally from below the medial commissure to the lateral commissure. On palpation, the swelling was soft, non-tender, and fluctuant, with no signs of blanching, redness, or warmth. The patient reported discomfort from the bulge beneath her right eye. There were no indications of internal damage such as scleral redness, burning sensation, itching, pain, or watery eyes. Mydriasis, partial external ophthalmoplegia, diplopia, and ptosis were present, along with an abnormal pupillary light reflex.

After removing the needle, the area was gently massaged for 10 to 15 seconds. The extraction procedure was postponed, and the patient was reassured and made comfortable. The parents were informed about the incident and reassured that there was no internal damage to the eye. Firm pressure was applied to the inferior orbital rim with a finger to prevent further swelling of the lower eyelid and to prevent the solution's entry into the infraorbital canal. An ice pack was applied to the infraorbital area for 20 minutes on and 10 minutes off, and the parents were advised to continue this application at home for two to three days [8]. They were also instructed not to apply heat for at least seven days following the procedure. The incident was documented in the patient's record, and the child was referred to an ophthalmologist for further evaluation.

**Figure 4:**
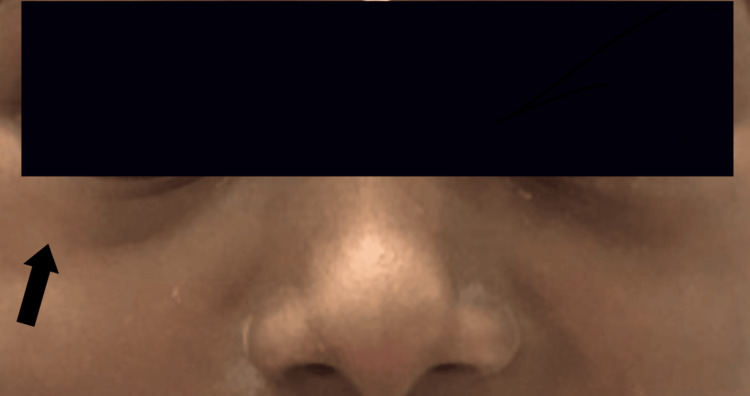
Extraoral photograph-with fully subsided infraorbital swelling-follow-up after 24 hours The arrow mark indicates the complete resolution of the infraorbital swelling after 24 hours, due to an inappropriate LA deposition technique LA: local anesthesia

The patient was prescribed atropine sulfate 0.1% (Jawa Pharmaceuticals India Pvt Ltd) eye ointment, for topical application, and Myatro 0.01% (Entod Pharmaceuticals Ltd., India) eye drop by the ophthalmologist. The patient was instructed to return for a follow-up the next day. During a phone review conducted four hours later, the patient’s mother reported a reduction in the swelling. When the patient returned the following day, the swelling in the infraorbital region had completely subsided (Figure 4). The patient reported no pain, loss of sensation, irritation, diplopia, or any other discomfort, and eye movements and reflexes appeared normal. The extraction procedure was rescheduled for one week later.

## Discussion

Ophthalmological complications from dental anesthesia can distress both pediatric patients and clinicians; while rare, they may be underreported, necessitating awareness of symptoms, pathophysiological mechanisms, and local anatomical pathways for optimal patient care [9].

The technique of administering LA plays a critical role in guiding the behavior of pediatric patients. Age-appropriate, non-threatening language, along with the use of distraction techniques, topical anesthetics, proper injection methods, and pharmacologic management, can help ensure a positive experience during LA administration. A thorough understanding of the gross and neuroanatomy of the head and neck is essential for accurate anesthetic placement and reducing the risk of complications [5]. In children, the vertical distance between the lower margin of the infraorbital foramen and the maxillary alveolar border is relatively less than in adults, making the infraorbital foramen more proximal to the maxillary alveolar border [10,11].

Jones et al. [12] concluded that infiltration is the preferred method for LA in children and that slow injections in small quantities are less painful. The use of aspirating syringes with lower gauge numbers and shorter lengths is preferred in children to enhance aspiration reliability and prevent deflection and needle breakage [13]. Needle insertion depth depends on the technique, patient age, and skull size. Penetration depth should be adjusted in children to avoid over-insertion risks [14].

In this case, an infiltration technique was employed utilizing a disposable 2 mL syringe (Dispovan, Hindustan Syringes & Medical Devices Ltd., India) with a higher gauge number and longer length and rapid deposition of the LA solution. An unanticipated movement of the child during the procedure caused the needle to deflect into the infraorbital region, leading to the deposition of the entire solution in the surrounding tissues. This resulted in increased discomfort and anxiety for the child.

Alamanos et al. [6] in a systematic review reported that 92% of ocular complications due to LA documented in the literature, including external ocular muscle palsies, were transient. Therefore, the existing evidence supports the resolution of diplopia. Notably, the resolution of transient complications typically occurred within a timeframe of six hours. In the current case, a telephonic review conducted four hours later indicated a reduction in swelling and a decrease in ocular symptoms.

Ocular complications resulting from dental LA are considered rare and typically mild in severity. The most frequently reported symptoms include diplopia, mydriasis, palpebral ptosis, and impaired abduction of the affected eye. The underlying mechanism is believed to involve the inadvertent diffusion of the anesthetic solution into the infraorbital region [9]. Ensuring proper anesthetic selection, accurate dosing, and precise technique is essential for the safe administration of LA, particularly in pediatric patients.

The differential diagnosis of LA allergy or hematoma was considered, but the absence of pain, swelling, and erythema indicated that the swelling was unlikely due to either cause, especially following an uneventful pulpectomy two days prior [15]. Comfortably positioning the patient allows the assistant to discreetly hand the syringe to the administrator, while distraction techniques, such as gentle cheek manipulation and maintaining conversation, can further reduce psychological stress and the risk of physical injury [14].

Managing a pediatric patient's fear and anxiety can be achieved by keeping the syringe out of sight and using protective stabilization. Protective stabilization is a critical component when managing children with special healthcare needs or behavioral challenges during dental procedures. It involves the use of physical methods, devices, or personnel to safely limit a patient's movement and prevent injury. This technique minimizes the risk of sudden, involuntary movements of the child that could lead to accidental harm. When used appropriately and ethically-with informed consent-protective stabilization can facilitate necessary dental care in a controlled and supportive environment, enhancing the overall effectiveness and safety of treatment [16].

## Conclusions

To effectively prevent complications during pediatric dental procedures, several critical factors must be considered. These include thorough case history taking, obtaining informed consent from the parent/legal guardian, and employing appropriate behavior management strategies tailored to the child. The use of suitable syringes and adherence to correct LA dosages are essential. Additionally, a comprehensive understanding of pediatric head and neck anatomy and careful monitoring of needle penetration depth are crucial in minimizing risk. In the event of a complication, immediate management steps should be taken. The procedure must be halted promptly, followed by a thorough evaluation and analysis of the underlying cause. The patient should be reassured, and ice packs should be applied to the affected area for approximately three minutes. If necessary, appropriate medications should be prescribed, and a follow-up should be scheduled to ensure proper recovery and resolution.

This case report details the management of a six-year-old female patient who presented with infraorbital swelling following LA administration during the extraction of a root stump. Despite initial swelling and symptoms suggestive of ocular complications, prompt intervention and follow-up led to complete resolution without lasting effects. This case underscores the importance of careful technique and patient management in pediatric dentistry to prevent and address potential complications.
